# Impact of Titanium Dioxide in the Mechanical Recycling of Post-Consumer Polyethylene Terephthalate Bottle Waste: Tensile and Fracture Behavior

**DOI:** 10.3390/polym13020310

**Published:** 2021-01-19

**Authors:** David Loaeza, Jonathan Cailloux, Orlando Santana Pérez, Miguel Sánchez-Soto, Maria Lluïsa Maspoch

**Affiliations:** Centre Català del Plàstic—Universitat Politècnica de Catalunya Barcelona Tech (EEBE-UPC), Av. d’Eduard Maristany, 16, 08019 Barcelona, Spain; alfonso.david.loaeza@upc.edu (D.L.); orlando.santana@upc.edu (O.S.P.); m.sanchez-soto@upc.edu (M.S.-S.); maria.lluisa.maspoch@upc.edu (M.L.M.)

**Keywords:** post-consumer opaque PET, titanium dioxide, recycling, physical aging, essential work of fracture

## Abstract

This work provides an experimental analysis regarding the fracture behavior of recycled opaque PET (rPET-O) containing titanium dioxide (TiO_2_) under plane stress conditions. For this purpose, a commercially post-consumer transparent colored/opaque PET flakes mix was processed using a semi-industrial extrusion calendering process. The manufactured rPET-O sheets had a TiO_2_ content of 1.45 wt.%. The mechanical and fracture properties of unaged and physically aged (1 year) samples were determined through uniaxial tensile experiments and the Essential Work of Fracture (EWF) methodology, respectively, and were compared to those of recycled transparent PET (rPET-T). Under tensile loading, independently of the aging time, rPET-O samples exhibited similar mechanical behavior as rPET-T up to the yield point. The main differences remained in the post-yielding region. The presence of TiO_2_ particles allowed reducing the strain energy density up to neck formation in aged samples. Regarding the EWF analysis, it is argued that the energy consumed up to the onset of crack propagation (w_e_) for rPET-T was mainly dependent of the molecular mobility. That is, the w_e_ value decreased by 26% when rPET-T was physically aged. Interestingly, w_e_ values remained independent of the aging time for rPET-O. In fact, it was highlighted that before crack propagation, the EWF response was principally governed by matrix cavitation ahead of the crack tip, which allowed a significant release of the triaxial stress state independently of the molecular mobility. This property enabled rPET-O to exhibit a resistance to crack initiation 17% higher as compared to rPET-T when the material was physically aged. Finally, independently of the aging time, rPET-O exhibited a resistance to crack growth approximately 21% larger than rPET-T due to matrix fibrillation in large scale deformation.

## 1. Introduction

According to recent statistics compiled by plastic Europe, in 2018, the packaging industry made up 39.9% of the total European plastics demand, thus representing the largest end-use plastic market with 20.4 million tons. The ongoing challenges related to end-of-life options and litter management for post-consumer plastics packaging waste is currently addressed through the European Union circular economy policy and its related plastics strategy which ranks recyclability and the uptake of recycled materials as the main priority [1].

Among all polymers used in the packaging industry, polyethylene terephthalate (PET) is one of the most versatile due to its good thermal, mechanical, and barrier properties together with its outstanding transparency and high gloss. In 2018, the European demand of PET amounted to approximately 4.3 million tons, of which 96% account for main packaging uses: 70% is used for non-beverage and beverage bottles, 19% for trays, and 7% for flexible packaging [2,3,4]. With its principal use being bottles, it was estimated that a total of 3.4 million tons of PET bottles were introduced in the western European market in 2018, of which 63% were collected for recycling. Mechanical recycling of post-consumer PET bottles is a well-established system which shows the significance of this material in the circular economy. That is, fiber production is the dominant end-use market for recycled PET flakes (rPET) [5].

Since 2010, post-consumer opaque PET bottles have been introduced in the recycling schemes of transparent colored PET bottles. Opaque PET (PET-O) is commonly used for packing milk, oil, mayonnaise, or even fabric softener. The addition of titanium dioxide (TiO_2_) to PET allows to protect the content from UV radiation, minimize gas permeation, and also to save weight and bring a glossy white aspect to the bottles when other pigments are not used [6]. However, mechanical recycling issues have been reported when more than 15 wt.% of recycled opaque PET flakes (rPET-O) are mixed with recycled transparent colored PET flakes [7]. That is, the presence of low concentration levels of TiO_2_ retards PET strain hardening and stress-induced crystallization in the rubbery temperature range, which impedes fiber manufacturing [8]. Currently, the total amount of manufactured opaque PET is difficult to evaluate. However, the sharp increase in the production of PET-O since 2016 for the milk sector boosts the demand to find recycling strategies for this material.

Recycling and repurposing of plastic waste is of the utmost importance in the conservation of the environment. However, the other consideration for reducing their environmental impact is to minimize plastic waste generation due to premature failure of plastic parts during their service life when they are uptaken to second-market application. In the past decades, the fracture behavior of virgin PET has been largely investigated under plane stress conditions through the essential work of fracture (EWF) [9,10]. This was argued by the large-scale yielding ahead of the crack tip prior to crack propagation. This concept postulates that the total work of fracture (*W_f_*) dissipated in a pre-cracked body could be split into two additive terms; the essential work required to generate a new free surface during loading (*W_e_*) and the non-essential or plastic work consumed by various deformation mechanism in the plastic zone surrounding the crack propagation plane (*W_p_*) [11,12]. As the former is surface-related and the latter volume-related, the following equations can be written,

(1) $$W_{f}=W_{e}+W_{p}=\;w_{e}lt+\beta w_{p}l^{2}t$$

(2) $$\frac{W_{f}}{lt}=w_{f}=w_{e}+\beta w_{p}l$$

where *w_f_* is the specific work of fracture, w_e_ the specific essential work of fracture (per ligament area), and *w_p_* the specific non-essential work of fracture (per volume unit). *l* is the ligament length, t the sample thickness, and *β* a shape factor correlated with the form of the plastic zone. In other words, *w_e_* and *βw_p_* indicate the resistance to crack initiation and propagation, respectively. They can be easily obtained from a linear regression of a set of values represented in a graph plotting *w_f_* versus *l* when experiments are performed under plane stress conditions.

According to the literature, the fracture behavior of PET mainly depends on the physical and hygrothermal aging process of amorphous material [13,14,15], the molecular orientation [12,16,17,18], the molecular structure [19], as well as the testing speed and temperature [16,20]. As opaque PET includes submicronic TiO_2_ particles (mean diameter = 261 nm [21]), it is expected that their well reported poor interfacial adhesion with the polymeric matrix affects the fracture parameters of PET. However, to the best of the author’s knowledge, there is no particular information available on the fracture properties of rPET-O neither under plane stress or plane strain conditions. Consequently, the main purpose of the present work is an experimental study regarding the impact of TiO_2_ on the crack propagation processes in post-consumer PET bottle waste. In addition, as physical aging has a profound impact on the PET fracture behavior, the mechanical and fracture properties were determined on unaged samples and samples physically aged for 1 year. In the present work, EWF experiments were conducted under plane stress conditions on thin calendered sheet processed using a semi-industrial extrusion-calendering process.

## 2. Materials and Methods 

### 2.1. Materials

**Figure 1:**
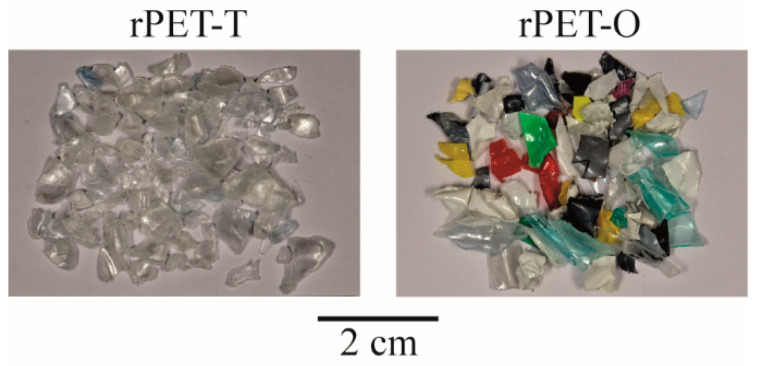
Post-consumer raw materials used in this study.

In this communication, two commercially recycled PET (rPET) grades reclaimed mainly from post-consumer containers were used as raw materials. Transparent rPET bottle grade flakes (referred to as rPET-T) were provided by a Spanish waste treatment company (PET Recuperaciones de Plásticos de Barcelona). A physical mix of transparent colored rPET with opaque rPET flakes (referred to as rPET-O) was kindly supplied by Suez (Bayonne, France) under the trade name Floreal. The visual aspect of the received post-consumer flakes of both materials can be seen in Figure 1.

### 2.2. rPET Sheet Manufacturing

A two-step process was used to manufacture rPET sheets. Prior to each processing step, rPET materials were dried for 4 h at 120 °C in a PIOVAN hopper-dryer (DSN506HE, Venice, Italy) with a dew point of −40 °C. During both processing stages, a N_2_ blanket was introduced in the feeding zone of the co-rotating twin-screw extruder Collin Kneter 25 × 24D (COLLIN GmbH, Ebersberg, Germany) having a screw diameter of 25 mm (L/D = 36) and seven heating zones.

In a first step, both rPET-T and rPET-O flakes were homogenized in order to obtain regular pellets (allowing a constant feeding in the second step). The effect of the temperature profile on the molecular weight (MW) degradation was initially investigated only using rPET-T. The extruder was operated using the temperature profiles compiled in Table 1 at a screw speed of 80 rpm. Based on intrinsic viscosity experiments, a less severe molecular degradation was achieved using the low temperature profile condition, as it will be explained in Section 3. Consequently, rPET-O was processed applying these optimum processing conditions. The extrudates were water-cooled, pelletized, and crystallized in a convection oven at 120 °C for 4h. Based on calcination analysis following the ISO-3451-1 standard [22], rPET-O pellets had a TiO_2_ content of 1.45 ± 0.05 wt.%. Samples were randomly collected throughout the whole homogenization step.

**Figure 2:**
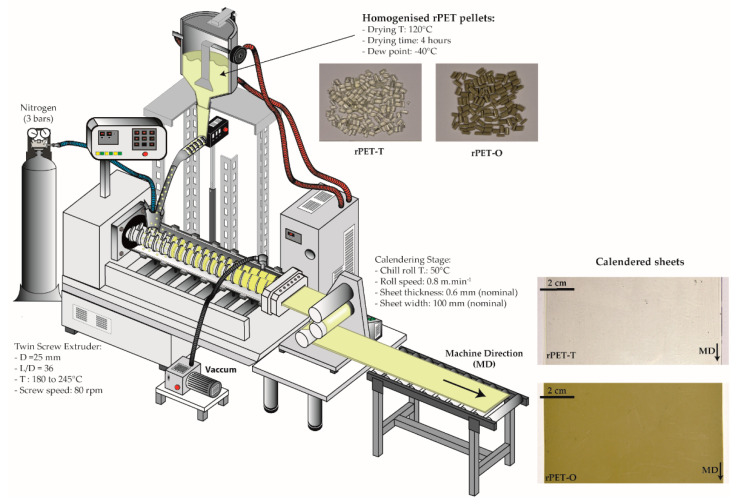
Schematic representation of the extrusion-calendering process of rPET sheets.

In a second step, 30 m of calendered sheets (nominal width: 100 mm; nominal thickness: 0.6 mm) of each homogenized rPET material were calendered (Techline CR72T, COLLIN, Ebersberg, Germany). The extruder was operated using the low-temperature profile conditions reported in Table 1. The chill roll temperature was set to 50 °C and the take-up speed was set to 0.8 m.min^−1^ in order to minimize molecular orientation. A schematic representation of the extrusion-calendering process of rPET sheets is depicted in Figure 2.

### 2.3. Molecular Characterization

The intrinsic viscosity, [*η*], was determined according to ASTM D2857 standard using an Ubbelohde 1B type viscometer. Prior to dissolution, samples were dried overnight at 120 °C under vacuum. Then, they were dissolved in a 60/40 phenol/1,1,2,2-tetrachloroethane solution at 110 °C for 30 min. After complete dissolution, the solutions were cooled to 25 °C, filtered, and tested. [*η*] (dL·g^−1^) was determined from the flow time of the pure solvent, t_0_ (s), and 4 polymer solutions with the following concentrations: 0.8, 0.6, 0.4, and 0.2 g·dL^−1^.

According to the solvent and temperature used in this study, the following Mark–Houwink relations can be found in the literature in order to calculate the number-average molecular weight, *M_n_* (g·mol^−1^), and the weight-average molecular weight, *M_w_* (g·mol^−1^), from [*η*] [23]:

(3) $$\left[\eta \right]=3.72*10^{-4}{\left(M_{n}\right)}^{0.73}$$

(4) $$\left[\eta \right]=4.68*10^{-4}{\left(M_{w}\right)}^{0.68}$$

### 2.4. Differential Scanning Calorimetry (DSC)

DSC experiments were performed on a MDSC Q2000 instrument (TA Instruments, New Castle, DE, USA) under a dry N_2_ atmosphere. Five to six milligrams of the samples was sealed in standard aluminum pans and subjected to a heating/cooling/heating procedure from 20 to 300 °C at a rate of 10 °C·min^−1^. The glass transition temperature (T_g_), the cold crystallization temperature (T_cc_), the crystallization temperature (T_c_), and the melting temperature (T_m_) were obtained from all thermal cycles. From the first and second heating, the degree of crystallinity, *X_c_*, was calculated as

(5) $$X_{c}=\frac{\Delta H_{m}-\Delta H_{cc}}{\Delta H_{m}^{0}*\emptyset_{PET}}$$

where Δ*H_m_* is the melting enthalpy (J·g^−1^), Δ*H_cc_* the cold crystallization enthalpy (J·g^−1^), Δ*H_m_*^o^ the melting enthalpy for a 100% crystalline *PET* (140 J·g^−1^ [24,25]), and ∅*_PET_* the weight fraction of PET.

### 2.5. Mechanical and Fracture Characterization

Based on DSC experiments, results suggested that both calendered rPET sheets exhibited a low percentage of crystallinity (7%), as it will be explained in Section 3.2. Consequently, the mechanical and fracture behavior of both rPET materials was assessed on samples (i) tested 1 day after processing (referred to as unaged) and (ii) stored at room temperature (RT) for 1 year (referred to as physically aged) in order to drive the physical aging process to equilibrium.

#### 2.5.1. Tensile Tests

Uniaxial tensile experiments were performed at different extension rates in order to compare the strain rate dependence of the mechanical behavior of rPET-O to the one of rPET-T. After processing, according to ISO 527-3 [26] type V dumbbell specimens were extracted from the center of the calendered sheets parallel to the machine direction (MD) with the following dimensions: overall length L = 115 mm, distance between grips L_0_= 80 mm, slender section width W = 6 mm, and nominal thickness t = 0.6 mm. The uniaxial tensile behavior was assessed using a universal testing machine (SUN 2500, GALDABINI, Cardano al Campo, Italy) equipped with a 1 kN load cell at RT (23 °C). The Young’s Modulus, E, was determined using a video extensometer (OS-65D CCD, Minstron, Taipei, Taiwan) using an initial length between marks in the slender section L_1_ = 25 mm. From the engineering stress–strain curves, the yield stress, σ_y_, was determined as the maximum stress. In order to determine the strain at the onset of necking, ε_n_, the strain rate was determined by deriving the extensometer strain by the testing time and ε_n_ was determined at the point where the strain rate starts to increase after yielding. The strain energy density up to the onset of necking, U_n_, corresponds to the area below the engineering stress–strain curve up to the onset of necking. All reported values are averages of five valid specimens tested at a constant crosshead speed of 1, 5, 10, and 20 mm·min^−1^.

#### 2.5.2. Fracture Behavior

**Figure 3:**
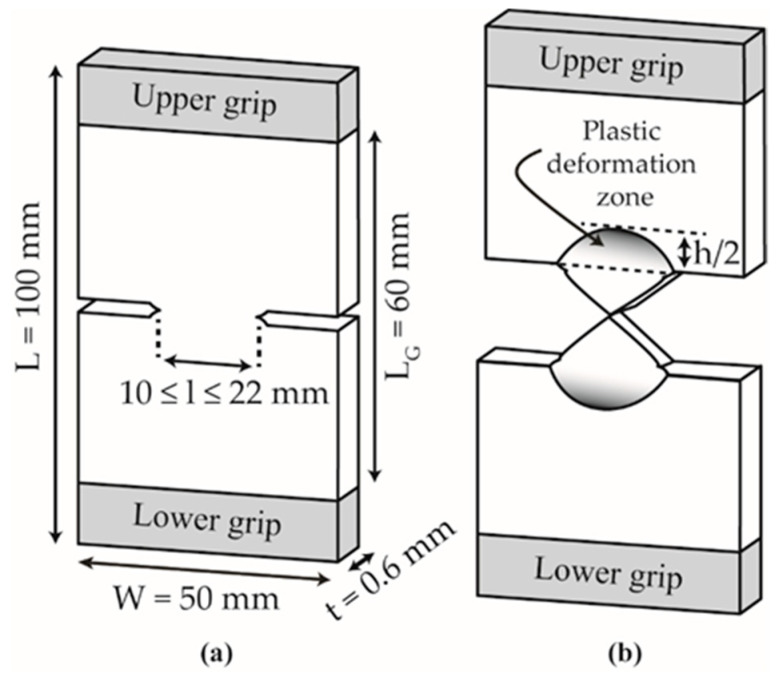
Schematic representation of the DDENT geometry with sample dimensions (**a**) before and (**b**) after testing.

For the EWF study, mechanical experiments and deformation measurements were performed using identical devices as described in Section 2.5.1. Experiments were performed at RT and at a constant crosshead speed of 10 mm·min^−1^. Deeply Double-Edge Notched Tensile (DDENT) samples were extracted from the center of the sheet, parallel to MD with the following dimensions: overall length L = 100 mm, distance between grips L_G_ = 60 mm, width W = 50 mm, and thickness t = 0.6 mm. A DDENT sample is shown in Figure 3a. Five ligament lengths, *l*, between 10 and 22 mm with a step of 3 mm were tested and repeated three times. Before testing, initial cracks were sharpened with a fresh razor blade. The total work of fracture, W_f_, was calculated by integrating the area below the load–displacement curves for each tested ligament length.

After testing, a binocular lens microscope (Carton, Pathumthani, Thailand) was used to determine the real ligament lengths and the height of the plastic deformation zone, h, surrounding the fracture zone, as shown in Figure 3b. A video monitoring system (Schneider Kreuznach, Bad Kreuznach, Germany) coupled to an optical strain measuring system (ARAMIS, GOM GmbH, Braunschweig, Germany) was used for an advanced strain analysis using digital image correlation (DIC).

### 2.6. Fractography Characterization

Scanning electron microscopy observations were performed on a JOEL-JSM5610 microscope (JOEL Ltd., Tokyo, Japan) in order to inspect the fractured surface of the DDENT samples. Observations were performed under vacuum with an accelerating voltage of 10 kV. Prior to observations, samples were sputter coated with a thin gold layer.

## 3. Results

### 3.1. Intrinsic Viscosity Measurements of Unprocessed Flakes and Homogenized Pellets

Initially, the effect of the temperature profiles compiled in Table 1 on the MW of rPET-T was investigated using dilute solutions. That is, intrinsic viscosity measurements were performed on both the unprocessed rPET flakes and the homogenized rPET-T pellets in order to evaluate the loss of MW induced by the exposure to high temperatures during processing. In fact, independently of the temperature profile used, the residence time into the extruder was found to be roughly similar (≈5 min). Therefore, the main objective was to optimize the processing temperatures in order to minimize the well-known thermal and hydrolytic degradations induced to post-consumer PET during mechanical recycling in order to guarantee the quality of the recycled material in second-commercial applications [27,28]. Additionally, the number-average MW, M_n_, and the weight-average MW, M_w_, were calculated using Equations (3) and (4). All the obtained results are reported in Table 2.

The intrinsic viscosity determined for unprocessed rPET-T flakes is in line with values reported in the literature for bottle grade PET [25,29,30]. In the present work, the commonly employed temperatures to process PET in industrial settings were mimicked through the high temperature profile reported in Table 1. Results clearly revealed a significant decrease in [*η*] (−23%) as compared to unprocessed flakes. This drop in the MW parameters is the result of the well reported chain-scission and main thermal degradation mechanisms that take place when PET is processed at high temperatures [31,32]. It equally highlighted the need to lower the processing temperatures in order to preserve the IV value. Accordingly, rPET-T flakes were processed using the medium and low temperature profiles. As expected, by decreasing the processing temperatures, the degree of molecular degradation gradually decreased. Considering these results, rPET-O flakes were homogenized using the low temperature profile. According to the literature, one of the minimum requirements that rPET should meet in order to be reprocessed properly is a [*η*] > 0.7 dL·g^−1^ [32,33,34]. The IV of homogenized rPET-O pellets using the low temperature profile decreased by 8% as compared to the unprocessed flakes. Accordingly, this result suggests a satisfactory preservation of the MW for its suitability in a recycling plant. Visually, the melt also exhibited a stable and uniform color. This observation was also remarkable as the content of fed flakes exhibiting different colors was not controlled.

As the impact of TiO_2_ on the mechanical and fracture behavior of rPET is of interest, from now on only rPET-O and rPET-T calendered sheets processed using the low-temperature profile (Table 1) will be considered for the sake of clarity. The IV were found to be stable between both materials after extrusion-calendering, with a level of 0.696 and 0.687 dL·g^−1^ for rPET-O and rPET-T, respectively. Initially, DSC tests were carried out to investigate the thermal behavior of the manufactured sheets, product of the extrusion-calendering process. Then, a comparative study was carried out to investigate the effect of the current content of TiO_2_ on the rPET thermal behavior. Table 3 summarizes the DSC data obtained from the thermal protocol described in Section 2.4.

### 3.2. Thermal Properties of the rPET Calendered Sheets

**Figure 4:**
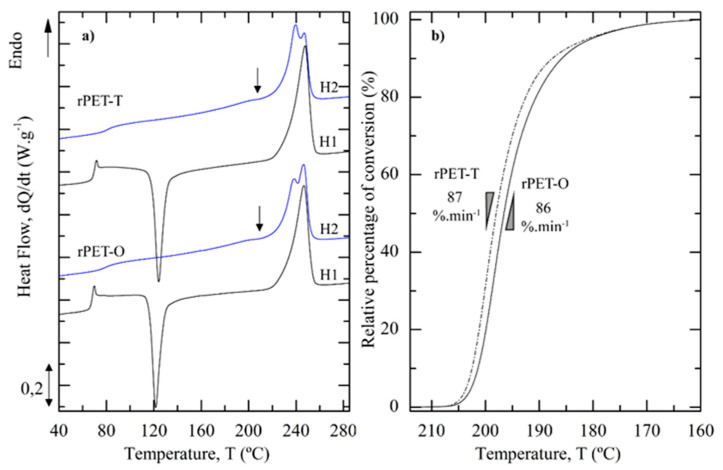
(**a**) First (H1) and second (H2) DSC heating scans at 10 °C·min^−1^ for rPET-T and rPET-O calendered sheets (Endo: direction of the endothermic transitions). The black arrow indicates the first melting peak in the second heating scans. (**b**) Relative percentage of conversion as a function of the temperature upon cooling at 10 °C·min^−1^. The maximum rate of conversion in %·min^−1^ is indicated for each sample.

According to the first heating scans (Figure 4a), rPET-O exhibited similar thermal properties as rPET-T. That is, even though T_g_, T_cc_ and T_m_ of rPET-O shifted to slightly lower temperatures, no significant differences were observed between both materials. Prior to the main melting peak, the DSC curve of both materials presented an exotherm (T_cc_ ≈ 122–124 °C) associated with the cold crystallization process. After subtracting the enthalpy associated to the cold crystallization process from the melting one, results revealed that the relative fast cooling rate applied to the materials at the die exit led to the manufacturing of sheets with a low degree of crystallinity (around 7%).

The cooling curve (not shown) of rPET-O showed a well-defined crystallization peak at T_c_ = 198 °C, identical to the cooling behavior of rPET-T. This indicates that both materials exhibited a high crystallization rate, characteristic of rPET materials [35,36]. While the onset of the crystallization process shifted to slightly lower temperatures for rPET-O (−2 °C as compared to rPET-T), its maximum rate of conversion (Figure 4b) remained similar to rPET-T. Upon constant cooling, this behavior led to a similar X_c_ between both materials, as reported in Table 3.

During the second heating scan (Figure 4a), the T_g_ of both materials showed a clear increase as compared to the first heating cycle. In fact, the large crystalline fraction developed on cooling (34%) significantly increases the energy required to increase the mobility of the amorphous regions and therefore shifts the glass transition towards higher temperatures. The cold crystallization process disappeared and a multiple melting behavior can be observed for both materials. That is, a small endotherm (indicated by an arrow in Figure 4a) was detected prior to the prominent double melting peak, which featured two overlapped endotherms. This melting behavior is in line with the literature [35,37,38,39,40] and ascribed to the melting of crystals formed during secondary and primary crystallizations, respectively, and to thickened lamellae formed as a result of the melt-recrystallization process on heating. In the present study, the main difference was in the intensity of the second and third endotherms in order of increasing melting point. Interestingly, in rPET-O, results suggested that the intensity of the highest temperature endotherm grew at the expense of the second one as compared to rPET-T. This result suggests that a larger fraction of thicker lamellas was probably formed as a result of recrystallization on heating in rPET-O as compared to rPET-T.

### 3.3. Mechanical Behavior

**Figure 5:**
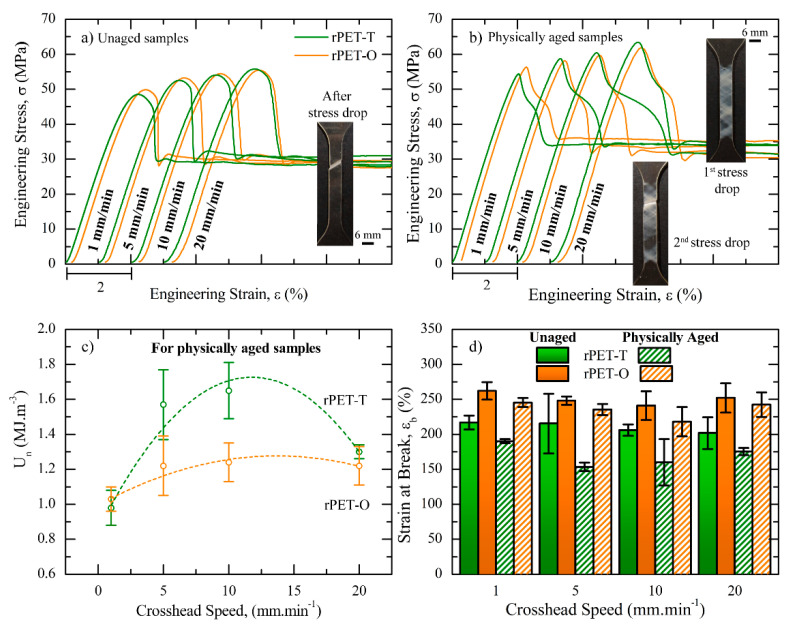
Effect of the crosshead speed on the mechanical properties of unaged and physically aged rPET-T and rPET-O: (**a**,**b**) engineering stress–strain curves; (**c**) strain energy density value up to the onset of necking versus crosshead speed, and (**d**) strain at break values versus crosshead speed.

Representative tensile engineering stress–strain curves of unaged and physically aged rPET materials are shown in Figure 5a,b, respectively. The resulting average Young’s modulus, E; yield stress, σ_y_; strain at the onset of necking, ε_n_; and strain energy density up to the onset of necking, U_n_, are reported in Table 4.

For both a given testing speed and storage time before testing, results suggested that rPET-O exhibited similar E and σ_y_ values than rPET-T, taking into account the experimental error involved. Independently of the storage time before testing, σ_y_ values gradually and similarly increased for both materials with increasing the testing speed. On the other hand, when the samples were physically aged for 1 year, the σ_y_ values increased around 12% as compared to unaged samples for a given testing speed. It is well known that the mechanical behavior of polymers depends on the molecular mobility. That is, while increasing testing speed decreases the macromolecular response time, physically aging of glassy polymers below T_g_ induces molecular packing, thus decreasing the free volume [41]. Both physical effects induce a significant decrease in the ability of the chain segments to dissipate energy through molecular motions and lead to an increase in σ_y_ values.

Regarding unaged samples, the instantaneous yield drop, ascribed to the well-established strain softening mechanism, gave rise to a localized neck, as shown in Figure 5a [42,43,44]. Drastic changes in the post-yielding behavior were observed when samples were physically aged at RT for 1 year. In fact, rPET-T samples exhibited two successive stress drops after yielding. During testing, visual observations revealed that the first stress drop were ascribed to the formation of multiple micro-shear bands over the whole slender section length, while the second stress drop corresponded to necking inception, as shown in Figure 5b. This behavior has already been reported in the literature for oriented glassy PET fibers as well as for rPET [45,46] and ascribed to the chain ability to be highly oriented as well as stretched upon tensile loading in order to initiate neck formation. Accordingly, the post-yielding behavior of physically aged rPET-T material can be plausibly explained as follows. At the yield point, the reduced molecular mobility is likely to induce a strong localization of chain segment orientation in multiple points in order to release the triaxial stress state, thus allowing the formation of multiple micro-shear bands. When the induced deformation reached a critical threshold, the presence of the previously formed shear bands acted as structural defects and promoted the onset of necking.

With increasing the testing speed up to 10 mm·min^−1^, results suggested that the onset of necking was gradually delayed to a larger engineering strain (c.f. Table 4); therefore, gradually increasing the strain energy density was required to yield the onset of necking (c.f. Figure 5c). A detailed inspection of the slender section during testing revealed the formation of a larger proportion of micro-shear bands with increasing testing speed, thus increasing the induced deformation before reaching the critical threshold for neck formation. At 20 mm·min^−1^, this behavior seemed to be inverted. These results can be attributed to an adiabatic transformation of plastic work into thermal energy, therefore increasing chain mobility and promoting necking. 

This scenario changed substantially when physically aged rPET-O samples were considered. The energy required to yield the onset of necking was lower as compared to rPET-T and remained fairly independent of the crosshead speeds from 5 to 20 mm·min^−1^. In other words, in rPET-O, the strain lag along the strain axis between the yield point and the onset of necking remained constant as reported in Table 4. It is important to recall that opaque PET contains submicronic TiO_2_ particles, which usually exhibit a poor interfacial adhesion with the polymeric matrix [21]. Accordingly, the observed behavior was likely to be due to TiO_2_ particles acting as larger structural defects than the generated shear bands, therefore providing an early onset of necking and lowering the strain energy density up to the onset of necking. 

As shown in Figure 5d, unaged and physically aged samples showed the typical behavior of ductile polymers with stable neck propagation up to 100% of their initial length owing to the cold drawing process. Independently of the testing speed and for a given material, strain at break values decreased over aging time as a result of the reduced chain ability to be highly oriented and stretched during stable neck propagation. 

### 3.4. Fracture Behavior

Before investigating the fracture properties, the validity of the EWF measurements should be verified. The ESIS-TC4 protocol [11] highlights the fulfillment of three basic requirements to validate the EWF concept: (i) the full ligament yielding prior to the onset of crack growth, (ii) the geometric similarity of the load–displacement curves between samples having different ligament lengths, and (iii) the pure plane-stress state condition. 

**Figure 6:**
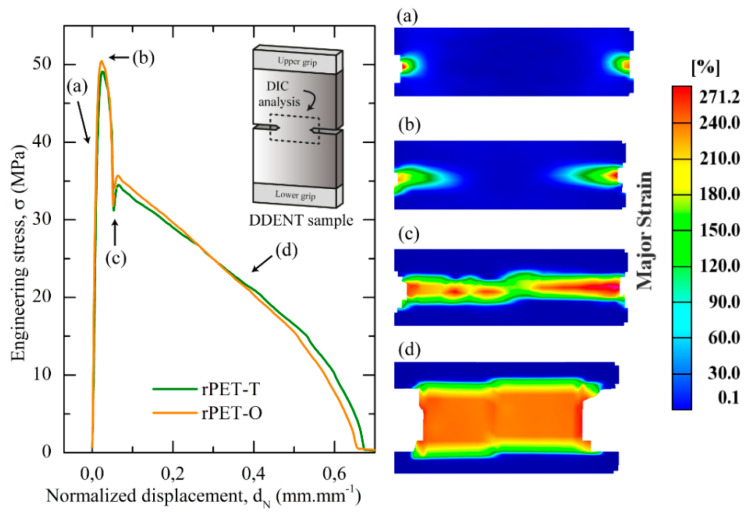
Engineering stress-normalized displacement curves for unaged DDENT samples tested at 10 mm·min^−1^. The displacement was normalized by a ligament length of 16 mm. Digital image correlation (DIC) analysis allowed to determine the strain field surrounding the ligament zone in some determined points.

**Figure 7:**
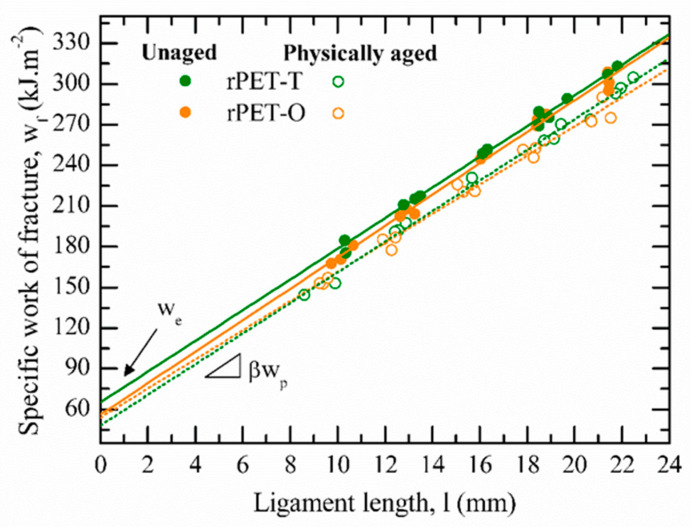
Specific work of fracture as a function of the ligament length for rPET-T and rPET-O materials. The points are experimental data and the lines represent experimental data fits according to Equation (2).

Figure 6 displays the engineering stress versus the normalized displacement curves for unaged rPET-T and rPET-O samples. It is noteworthy that after yielding, an instantaneous stress drop was observed for both materials. A similar phenomenon has been reported for amorphous copolyesters [19,47] and corresponds to the full ligament yielding of the DDENT samples. In this study, this hypothesis was carefully examined using DIC analysis as shown by the reported strain fields in Figure 6. It is important to note that the yielding process initiated from both notches in point a. That is, well before the stress maximum (point b) as a result of the high stress concentration induced by both sharpened notches. Before crack propagation, the expected full plastic collapse of the ligament was observed in point c, which fulfilled the abovementioned first basic criterion of the EWF concept. Similar observations were obtained for physically aged samples, therefore for the sake of clarity, results are not shown. The self-similarity requirement between load–displacement curves was verified during experiments for all the samples. Finally, in Figure 7, the linear increase in the specific work of fracture with increasing ligament length confirmed that measurements were performed under pure plane stress conditions. Accordingly, all basic requirements were satisfied and the EWF concept could be applied in order to compare the fracture toughness of both post-consumer unaged and physically aged PET materials.

**Figure 8:**
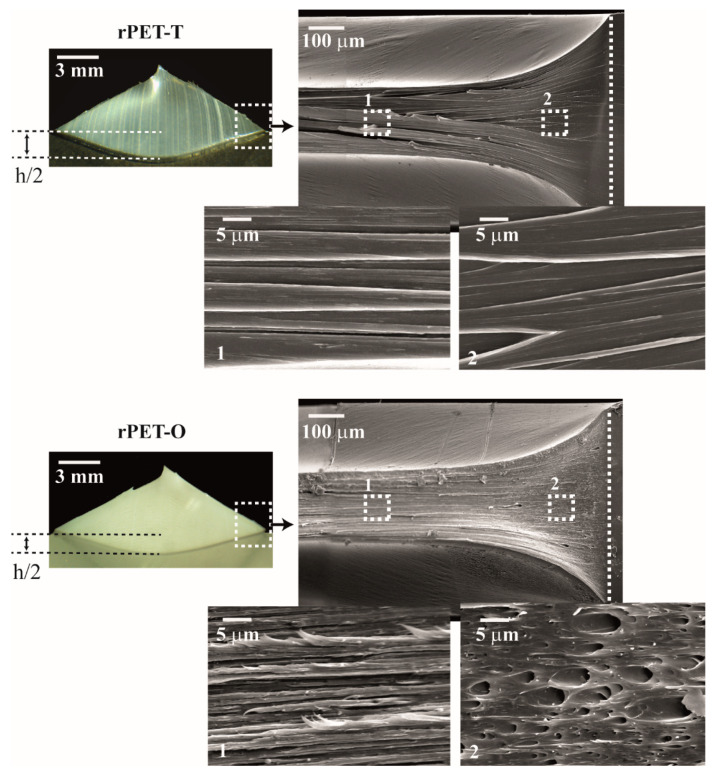
Micrographs taken from the plastic deformation zone of the broken unaged DDENT samples. h corresponds to the height of the plastic deformation zone. The fractured surface ahead of the crack tip (white dashed rectangle) was observed by SEM. The white dashed line indicates the initial crack tip.

Table 5 lists the EWF parameters of unaged and physically aged rPET-T and rPET-O materials obtained at a crosshead rate of 10 mm·min^−1^. For both unaged materials, the high values obtained for both w_e_ and βw_p_ terms are characteristics of tough polymers, as visually confirmed in Figure 8. SEM observations of the fractured surface of unaged DDENT specimens revealed that rPET-T failed by ductile tearing. In contrast the plastic behavior of rPET-O seemed to be governed by different deformation mechanisms. That is, microvoiding is observed in a restricted region at the tip of the notches that in turn triggers latter matrix fibrillation.

The results of EWF tests showed that in unaged rPET-T, the energy needed to initiate crack propagation (represented by w_e_) is 14% higher than for rPET-O. Interestingly, no significant differences were found in the volumetric plastic work (βw_p_, also referred to as the energy dissipated in the plastic zone) between both materials. In the present study, unaged rPET-T exhibited higher w_e_ values than those most commonly reported in the literature for unoriented glassy PET specimens tested at RT (from 35 to 56 kJ/m^2^ [9,14,15,19,48,49]). Such an increase is likely to be induced by the low degree of crystallinity of the tested DDENT samples (X_c_ = 7%, Table 3), which may act as morphological reinforcement and apparently enhanced the resistance to crack initiation (leading to higher w_e_ value). As rPET-T and rPET-O specimens exhibited similar X_c_ values (Table 3), the lower w_e_ data exhibited by the latter may be ascribed to the matrix cavitation process taking place ahead of the crack tip which lowers the resistance to crack initiation. 

As w_p_ cannot be directly calculated from Equation (2) due to its combination with the shape factor β, a detailed analysis of the βw_p_ term was necessary to evaluate the specific non-essential work of fracture. Following the method proposed by the ESIS-TC4 protocol [11], the necked zone of the broken unaged DDENT samples was visually inspected, as shown in Figure 8. Among the different plastic deformation zone geometries proposed in the literature [11,50], a parabolic-like form was present in both materials. Accordingly, the height of the plastic zone was determined for each tested ligament length (c.f. Figure 3 and Figure 8). Thus, one can estimate β by h = 1.5^×^β^×^l and from the best linear regression of a set of values represented in a graph plotting h versus l. 

In Table 5, the similarity in the plastic term, βw_p_, between both unaged materials can be seen when the competition between β and w_p_ data is discussed. Over the range of data considered, results suggest that unaged rPET-O specimens exhibited a lower β value than rPET-T ones. This reduction might indicate that the presence of a low content of TiO_2_ particles imposed physical constraints on the polymeric matrix, thus limiting the volume of polymer involved in plastic deformation outside the fracture surface. Nevertheless, the plastic energy absorbed per unit volume in rPET-O increased by 20% as compared to rPET-T. This increase in the w_p_ term could be justified considering that the tearing contribution considerably increased during fracture, which in turn enhanced the resistance to crack growth. Thus, rPET-O presented a similar βw_p_ value as compared to rPET-T because the noticeable increase in the work dissipated in the plastic zone (w_p_) was counterbalanced by a slight decrease in the volume involved in large-scale deformation outside the fracture process zone. 

When physically aged samples are considered, the above-mentioned SEM observations regarding the fractured surfaces of DDENT samples remained similar (not shown for the sake of clarity). The shape factor, β, as well as the work dissipated in the plastic zone, w_p_, exhibited similar trends as the unaged samples. Over aging time, the reduced molecular mobility limited the volume of polymer involved in large scale deformation (lowered β) and enhanced the work consumed to plastically deform the polymer (higher w_p_). The main difference between both rPET materials remained in the energy needed to initiate crack propagation, w_e_. While rPET-T exhibited a strong deterioration in w_e_, this parameter seemed to remain unaffected in rPET-O. This result highlighted that prior to crack propagation, the EWF response of rPET-O was principally governed by matrix cavitation ahead of the crack tip, which allowed a significant release of the triaxial stress state independently of the molecular mobility.

## 4. Conclusions

In this study, the mechanical and fracture behavior under plane stress conditions of a commercially available post-consumer transparent colored PET physically mixed with opaque PET flakes was evaluated. The obtained calendered sheets (referred to as rPET-O) exhibited a stable and uniform color with a TiO_2_ content of 1.45 wt.%. Under uniaxial tensile loading, unaged rPET-O exhibited the classical stress-strain behavior of ductile polyesters. When the molecular mobility decreased as a result of the physical aging process, TiO_2_ particles promoted necking and the energy density consumed to develop a localized neck remained independent of the testing speeds considered in this study. Regarding the Essential Work of Fracture analysis, it was highlighted that before crack propagation, the EWF response was mainly governed by matrix cavitation ahead of the crack tip which allowed a significant release of the triaxial stress state independently of the molecular mobility. Accordingly, the energy needed to initiate crack propagation remained constant over aging time.

## Figures and Tables

**Table 1:** Processing conditions considered in this study.

Temperature Profile	Temperature From Zone 1 to Die (°C)
High	200/240/255/260/265/270/270
Medium	190/230/245/250/255/260/260
Low	180/215/235/240/240/245/245

**Table 2:** Intrinsic viscosity at 25 °C and MW parameters for rPET-T and rPET-O unprocessed flakes and homogenized pellets, respectively.

Material	Temperature Profile	[η] (dL·g^−1^)	M_n_ (kg·mol^−1^)	M_w_ (kg·mol^−1^)	M_w_/M_n_
rPET-T flakes	-	0.875	41.5	64.8	1.56
rPET-T pellets	High	0.677 (−23%) ^1^	29.2	44.4	1.52
	Medium	0.717 (−18%) ^1^	31.6	48.3	1.53
	Low	0.790 (−10%) ^1^	36.1	55.7	1.54
rPET-O flakes	-	0.792	36.2	55.9	1.54
rPET-O pellets	Low	0.726 (−8%) ^1^	32.2	49.2	1.53

^1^ The degradation percentages arise from the difference between the measured IV of the corresponding unprocessed flakes and the homogenized pellets.

**Table 3:** Thermal properties of the manufactured rPET sheets.

	1st Heating				Cooling		2nd Heating			
Material	T_g_ (°C)	T_cc_ (°C)	T_m_ (°C)	X_c_ (%)	T_c_ (°C)	X_c_^1^ (%)	T_g_ (°C)	T_m1_ (°C)	T_m2_ (°C)	T_m3_ (°C)
rPET-T	70	124	248	7	200	34	81	204	240	247
rPET-O	69	122	247	8	198	34	79	204	239	246

^1^ X_c_ (%) = ΔH_c_/(ΔH_m_^0^*φ_PET_), where ΔH_c_ is the enthalpy of crystallization during cooling, φ the weight fraction of PET and ΔH_m_^0^ the melting enthalpy for a 100% crystalline PET (140 J·g^−1^) [24,25].

**Table 4:** Uniaxial tensile properties at different crosshead speed. For information purposes, T and O referred to as rPET-T and rPET-O materials, respectively. P.A. referred to as physically aged samples.

Property	Sample		Crosshead Speed (mm·min^−1^)			
			1	5	10	20
E(GPa)		T	2.1 ± 0.1	2.1 ± 0.1	2.2 ± 0.1	2.1 ± 0.2
		O	2.0 ± 0.1	2.1 ± 0.2	2.2 ± 0.1	2.1 ± 0.2
	P.A.	T	2.1 ± 0.1	2.2 ± 0.1	2.3 ± 0.2	2.2 ± 0.2
		O	2.2 ± 0.1	2.3 ± 0.1	2.3 ± 0.2	2.3 ± 0.2
$\sigma_{y}$ (MPa)		T	48 ± 4	53 ± 1	54 ± 1	56 ± 5
		O	50 ± 1	53 ± 1	54 ± 1	55 ± 2
	P.A.	T	54 ± 3	59 ± 5	61 ± 4	64 ± 2
		O	55 ± 1	58 ± 2	60 ± 2	62 ± 1
$\varepsilon_{n}$ (%)		T	2.7 ± 0.2	2.9 ± 0.1	3.1 ± 0.1	3.2 ± 0.1
		O	2.7 ± 0.2	2.9 ± 0.1	3.0 ± 0.1	3.0 ± 0.1
	P.A.	T	2.7 ± 0.2	4.0 ± 0.6	4.2 ± 0.4	3.4 ± 0.1
		O	3.0 ± 0.3	3.3 ± 0.4	3.3 ± 0.2	3.3 ± 0.2
$U_{n}$ (kJ·m^−3^)		T	0.9 ± 0.1	1.0 ± 0.1	1.0 ± 0.1	1.1 ± 0.1
		O	0.8 ± 0.1	1.0 ± 0.1	1.1 ± 0.1	1.0 ± 0.1
	P.A.	T	1.0 ± 0.1	1.6 ± 0.3	1.7 ± 0.2	1.3 ± 0.1
		O	1.1 ± 0.1	1.2 ± 0.2	1.2 ± 0.1	1.2 ± 0.1

**Table 5:** The fracture parameters of rPET-T and rPET-O materials at 10 mm·min^−1^.

Sample Nomenclature	w_e_ (kJ·m^−2^)	βw_p_ (MJ·m^−3^)	β (x10^−2^)	w_p_ (MJ·m^−3^)	R^2^ (w_f_ vs. l)
Unaged					
rPET-T	65 ± 4	11.3 ± 0.2	13.4 ± 0.3	84 ± 4	0.9945
rPET-O	56 ± 4	11.6 ± 0.3	11.5 ± 0.4	101 ± 7	0.9927
Physically aged					
rPET-T	48 ± 4	11.3 ± 0.2	11.1 ± 0.6	102 ± 1	0.9938
rPET-O	56 ± 5	10.5 ± 0.3	8.8 ± 0.4	126 ± 2	0.9895

## Data Availability

Data sharing is not applicable to this article.
